# NMR Spectroscopy: Molecular Insights into Cell Wall Collapse and Oxidative Stress of *Escherichia coli* Induced by Imidazole-Activated Eutectic Solvents

**DOI:** 10.1021/acsomega.6c01977

**Published:** 2026-06-09

**Authors:** Maurelio Cabo, Lexua Mclaughlin, Dennis LaJeunesse

**Affiliations:** Department of Nanoscience, Joint School of Nanoscience and Nanoengineering, 14616 University of North Carolina Greensboro, Greensboro, North Carolina 27455, United States

## Abstract

Antimicrobial resistance (AMR) presents a critical global health challenge, driving the search for unconventional antimicrobial agents. Despite increasing interest in deep eutectic solvents (DESs) as antimicrobial materials, the molecular determinants of their antibacterial activity remain insufficiently understood, particularly for gram-negative bacteria such as *Escherichia coli* with protective envelope barriers. This study investigates imidazole-activated DES to clarify how proton-active components influence solvent–cell interactions and bacterial inactivation mechanisms. Using complementary biological assays and NMR spectroscopy, imidazole was identified as the dominant bioactive component, functioning as a molecular activity switch within eutectic formulations. Minimum inhibitory concentration (MIC) measurements revealed strong bactericidal effects exceeding 90% cell death. Scanning electron microscopy showed distinct damage phenotypes, where DES induced cell wall collapse, while ternary DES (TDES) caused catastrophic wall–membrane rupture. Reactive oxygen species (ROS) assays demonstrated a pronounced oxidative surge, linking structural disruption to redox imbalance. ^1^H and COSY NMR analyses further revealed imidazole-driven perturbations in metabolite environments consistent with membrane destabilization and metabolic collapse. These findings establish imidazole-activated eutectic solvents as promising multimodal antimicrobial systems and highlight NMR spectroscopy as a practical platform for mechanistic antimicrobial and AMR-related investigations.

## Graphical abstract

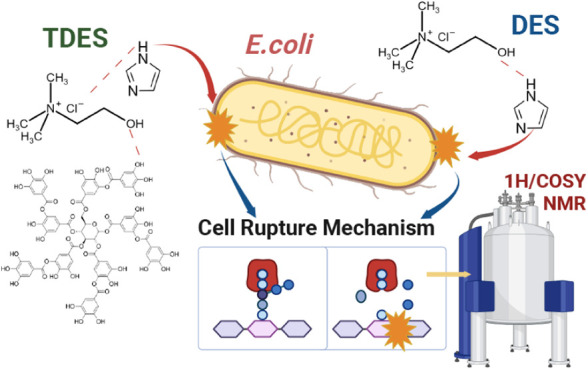

## 1 Introduction

Over the next 25 years, antimicrobial resistance is projected to cause up to 39 million deaths worldwide.^1,2^ This growing threat highlights the urgent need to explore new types of chemical systems that go beyond traditional antibiotic approaches. One promising and emerging class of antimicrobial agent materials is known as deep eutectic solvents (DESs).^3^ DESs are liquids formed through strong hydrogen-bond interactions between two components: a hydrogen-bond acceptor and a hydrogen-bond donor. An important advantage of these systems is that their properties can be easily adjusted, allowing researchers to modify key characteristics, including antimicrobial effectiveness.

This flexibility may influence several biological interactions, such as attachment to the cell surface,^4^ increased permeability of the cell envelope,^5^ disruption of ionic balance within the cell,^6^ and irreversible damage to essential cellular macromolecules.^7^ However, despite their chemical potentials, many DES formulations still present important research gaps. It remains unclear which components contribute most to antimicrobial activity and which analytical technique can help explain how and why these systems work. These questions are especially relevant for gram-negative bacteria such as *Escherichia coli*, whose outer membrane (OM) acts as a strong barrier that limits the entry of many antimicrobial agents.

Among the components used in deep eutectic solvents, imidazole-based systems have been recently studied because imidazole is a key structural element in many bioactive molecules, particularly those with antimicrobial activity.^8^ Imidazole is a heterocyclic aromatic compound with amphoteric character, meaning it can function both as a hydrogen-bond donor and a hydrogen-bond acceptor. It can also participate in important molecular interactions, such as π–π and cation−π interactions, and influence the local acidity or basicity of its surroundings.^9^ In biological systems, the imidazole ring plays a crucial role in the function of the amino acid histidine, which is involved in many enzymatic processes. Also, imidazole-containing eutectic liquids may interact more effectively with negatively charged bacterial surfaces, alter the organization of water near cell membranes, and affect proton or ion balance across the membrane. These effects may increase the vulnerability of gram-negative bacteria to membrane disruption and oxidative stress.

The relevant studies, for instance from Hu et al, found that using imidazole-based chloride ionic liquids (CnMIMCl), antibacterial activity against *Staphylococcus aureus* revealing that this agent enhances bacterial inhibition by promoting oxidative stress and membrane disruption, thus providing a tunable molecular framework for new antimicrobial design.^10^ In Moghadam et al.’s study, imidazole-containing 4-anilinoquinazoline derivatives were rapidly synthesized using a choline chloride:urea deep eutectic solvent (80 °C, 15–20 min), yielding higher efficiencies (60–72%), and biological assays identified compound 8k as highly potent (IC50 = 0.11 μM), with docking studies revealing critical imidazole-mediated interactions, highlighting imidazole’s importance as a bioactive scaffold and supporting sustainable drug design strategies.^11^ Furthermore, in Mjalli et al.’s study, a novel imidazole-based deep eutectic solvent composed of imidazole and monoethanolamine (MEA) was prepared by simple heating and stirring and characterized, revealing strong electrostatic hydrogen-bonding interactions, significant melting-point depression, and high fluidity, thereby highlighting imidazole’s key role as a tunable hydrogen-bonding component that advances the rational design of functional eutectic solvents for future bioactive and antimicrobial materials.^12^

In this study, we investigate two imidazole-enabled formulations—a deep eutectic solvent (DES) composed of choline chloride and imidazole and a ternary deep eutectic solvent (TDES) composed of choline chloride, imidazole, and tannic acid—against *Escherichia coli*. Rather than relying solely on conventional antibacterial assays, we employ NMR-based molecular spectroscopy to examine how imidazole functions within these eutectic systems. Our approach probes the hypothesis that imidazole does not merely coexist in the solvent matrix, but actively modifies the solvent microstructure, thereby strengthening interactions with the bacterial envelope, promoting membrane destabilization through distinct physical mechanisms, and enhancing oxidative stress to lethal levels that result in rapid cell death.

By integrating NMR spectroscopy with biological assays, we aim to add knowledge within a more efficient analytical framework for studying antimicrobial systems, potentially reducing the experimental workload, time, and cost associated with extensive laboratory testing. More broadly, this strategy may support the rational design, modification, and rapid characterization of bioactive solvent platforms. In the long term, such analytical methodologies could contribute to streamlined protocols that are relevant to both biomedical research and industrial applications.

## 2 Experimental Section

### 2.1 Materials

Yeast, Peptone, Dextrose, 2xYT Agar, LB Broth Miller, Tannic Acid, and Choline Chloride (99%) were purchased from Fisher Scientific (Thermo Fisher Scientific, Waltham, MA, USA). Imidazole (99%), and 2′,7′-dichlorodihydrofluorescein diacetate (H_2_ DCFDA) fluorescent dye was purchased from Sigma-Aldrich (Sigma-Aldrich, St. Louis, MO, United States). The chemicals utilized in this study were used in their original form without any purification.

### 2.2 Synthesis of Eutectic Solvents

In a 3:7 molar ratio,^13^ we mixed choline chloride (8.39 g) and imidazole (9.53 g) and heated them at 120 °C on a hot plate for 3 h with pre- and postheating of 1 h each since the hot plate is of an analog type (set at 4.5). The mixture turned into a yellowish liquid solution, which was then left to rest for 24 h in room temperature. An amount of tannic acid (TA), at 0.5% of the total volume of choline chloride/imidazole (ChCl/Imi) deep eutectic solvent (DES), was added the following day, and the liquid’s color transformed into a reddish-black shade.^14^ Table 1 shows the sample weights used and the computed final concentration.

**Table 1:** Sample Weight Used and Final Concentration

Samples	Weight (g)	DI Water Added (g)	Final Concentration (mg/mL)
Choline Chloride (ChCl)	8.39	9.53	22.20
Imidazole (Imi)	9.53	8.39	25.30
Tannic Acid (TA)	0.0767	17.92	0.202
Choline Chloride/Imidazole (DES)	17.92	0	55.70
Choline Chloride/Imidazole/Tannic Acid (TDES)	17.99	0	67.69

### 2.3 Plate–Hole Diffusion Assay

The 2 × YT agar was prepared in DI water according to the laboratory formulation guidelines by MacWilliams et al.^15^ The prepared agar was sterilized by autoclaving at 121 °C for 15 min. After sterilization, the molten agar was allowed to cool to room temperature and was then aseptically poured into sterile Petri dishes. The plates were left undisturbed until fully solidified and adequately dried. A wild-type strain of *E. coli* BW25113 suspension was prepared. Using a disposable plastic inoculum loop, the bacterial culture was evenly spread over the entire surface of the LB agar plate to produce a uniform lawn of growth. The inoculated plates were allowed to rest briefly at room temperature to permit the absorption of excess surface moisture.

A sterile punch, 7 mm in diameter, is used to create wells in the agar as per Hayhoe et al.’s protocol.^16^ The agar plugs are carefully removed and discarded, and the wells are labeled as required. 100 μL volume of samples is pipetted into each well. The plates are then left at room temperature for a prediffusion period to allow the substances to diffuse into the agar matrix. Following diffusion, the plates are incubated at 37 °C with shaking at 150 rpm over a 24 h period. After incubation, antimicrobial effects are assessed by measuring the diameter of any zones of inhibition surrounding the wells from the underside of the plate using the zone diameter interpretative standards for *E. coli* published by Hudzicki, also known as the Kirby-Bauer disk diffusion susceptibility test protocol.^17^

### 2.4 Minimum Inhibitory Concentration (MIC)

The microdilution assay was used to determine the minimum inhibitory concentration (MIC) of eutectic solvents by evaluating the visible growth of microorganisms in broth media. A wild-type strain of *E. coli* BW25113 was grown in Luria–Broth (LB) media at 37 °C in a 50 mL conical flask with shaking at 150 rpm prior to sample preparation. Cultures were grown to the mid-log phase at an optical density at 600 nm (OD_600_) of 0.5. OD_600_ measurements were made using a Thermo Scientific Nanodrop 2000C spectrophotometer. LB media (100 μL) was added to a 96-well round-bottom polystyrene microtiter plate (Costar, Corning Inc., Kennebunk, USA), followed by a series of 2-fold dilutions of individual components and eutectic solvents, based on the concentrations disclosed in Table 2. Then, 10 μL of inoculum was added to each well, followed by additional LB media to a final volume of 200 μL. LB media with inoculum but without eutectic solvents was used as a control. Bacterial growth in LB media with added eutectic solvents was obtained at 37°C with shaking at 150 rpm over a 24 h period. The absorbance measurements (OD_600_) were taken using the BioTek Cytation 5 Imaging Multimode Reader. Experiments were done in triplicates to ensure validity.

**Table 2:** Percent Cell Viability with Standard Deviation

Sample	Concentration (mg/mL)	% Cell Viability	Standard Deviation (SD)
ChCl	22.20	11.47	0.089
	11.10	–3.39	0.054
	5.55	2.69	0.11
	2.78	–2.89	0.089
	1.39	–0.78	0.090
Imi	25.30	93.16	0.022
	12.65	66.59	0.055
	6.33	48.45	0.041
	3.16	40.54	0.063
	1.58	7.36	0.021
TA	0.202	7.33	0.069
	0.101	14.60	0.020
	0.051	16.72	0.049
	0.025	12.11	0.043
	0.013	13.91	0.079
DES	55.70	94.57	0.004
	27.85	88.81	0.012
	13.93	65.24	0.320
	6.96	18.81	0.063
	3.48	–6.88	0.243
TDES	67.69	85.01	0.014
	33.85	91.08	0.002
	16.92	91.28	0.002
	8.46	91.31	0.002
	4.23	26.57	0.019

### 2.5 Reactive Oxygen Species (ROS) Assay

A ROS assay was performed to determine intracellular oxidation levels following the exposure of *E. coli* cells to eutectic solvent treatments. *E. coli* *cells* were cultured independently in 10 mL LB media. Experiments were initiated when the cell culture reached an optical density (OD_600)_ of 0.5. Bacterial cell pellets from media cultures were formed via centrifugation for 10 min at 3500 rpm. The cell pellets were washed with 1x Phosphate Buffer Saline (PBS), centrifuged again, and resuspended in 1x PBS. Following this, cell solutions underwent sonication to break the cells open and then were dyed with 2 μL (1:2000 ratio) of 10 mM 2′,7′-dichlorodihydrofluorescein diacetate (H_2_ DCFDA) fluorescent dye. The cells then underwent incubation at 37 °C with shaking for 30 min. Centrifugation was used to pellet the incubated cells, and they were rewashed with 1x PBS to remove any remaining H_2_ DCFDA. Next, 10 μL of individual components (choline chloride, imidazole, and tannic acid), DES, and TDES treatment was added to the *E. coli* cell solution along with 200 μL buffer in a 96 well plate and then incubated at 37 °C for 2 h. A 2-h time point was selected to reduce confounding from late-stage growth inhibition and cell death that can bias fluorescence-based ROS measurements. Untreated *E. coli* cells were used as a control. Fluorescence intensity for detecting ROS was measured using the BioTek Cytation 5 Imaging Multimode Reader at an excitation wavelength of 485 nm and an emission wavelength of 535 nm. The experiments were also done in triplicates to ensure validity.

### 2.6 Scanning Electron Microscopy (SEM)

The bacterial samples for SEM imaging using a JEOL JSM-IT800 FESEM were prepared using a modified fixation protocol based on standard procedures. A glutaraldehyde-formaldehyde mixture (Karnovsky’s fixative) was used for primary fixation to preserve cellular morphology, prepared with 1x phosphate-buffered saline (PBS). The cell pellet samples were taken after MIC and ROS treatments. After removing the supernatant, 200 μL of a prepared stock of glutaraldehyde-formaldehyde/phosphate-buffered saline (PBS) mixture was added to the cell pellet, centrifuged, and then allowed to sit for 30 min to remove excess fixative and eutectic solvents. The bacteria were then centrifuged and washed five times with autoclaved DI water. Centrifugation was employed during the rinsing and dehydration steps to ensure proper pelleting of bacterial cells. A low-speed centrifugation (3500 RCF for 10 min at room temperature) was used to avoid potential damage to the bacterial cells. A gradual dehydration series was performed using increasing concentrations of ethanol (25%, 50%, 75%, 95%, and 100%). Following the dehydration step, the bacterial suspension, 10 μL in volume, was deposited onto a clean Si wafer for further imaging and allowed to dry at room temperature for 24 h.

### 2.7 Nuclear Magnetic Resonance (^1^H and COSY)

The equipment used for the acquisition of the spectra was an Agilent 400 MHz equipped with a TB_MR1107W018 probe. Processing of spectra was carried out under the program MestReNova version 15.0.1 (Mestrelab Research, Santiago de Compostela, Spain). All experiments were performed at 25 °C. ^1^H NMR spectra were recorded between −2 ppm and 14 ppm, with a relaxation delay (d1) of 2 seconds and a number of scans (ns) of 64, resulting in a total acquisition time of 3 min and 30 s. This was followed by COSY NMR spectra, which were recorded with 16 scans per t1 increment and 256 t1 increments, resulting in a total acquisition time of 1 h and 22 min. Deuterium oxide (D_2_O) solvent was used for all the samples. Samples were taken after MIC and ROS assays, followed by centrifugation to separate cells from the media/solvent used. For cell pellet samples, 200 μL of D_2_O solvent was added into an Eppendorf tube (600 μL) followed by vortexing, and then transferred into NMR tubes to mix with 400 μL of D_2_O solvent.

## 3 Results and Discussion

### 3.1 Plate–Hole Diffusion Assay Analysis

To determine which of the individual components—choline chloride, imidazole, and tannic acid—can inhibit bacterial growth, a plate–hole diffusion assay was performed. The concentration of each component was selected based on our previous study. Each compound was dissolved separately in deionized (DI) water to prepare liquid samples. As shown in Figure 1, the zone of inhibition (ZOI) was used to evaluate antibacterial activity. Choline chloride, Figure 1A, and tannic acid, Figure 1C, produced no visible ZOI, indicating that these compounds do not exhibit antibacterial properties when used alone under the tested conditions.

**Figure 1:**
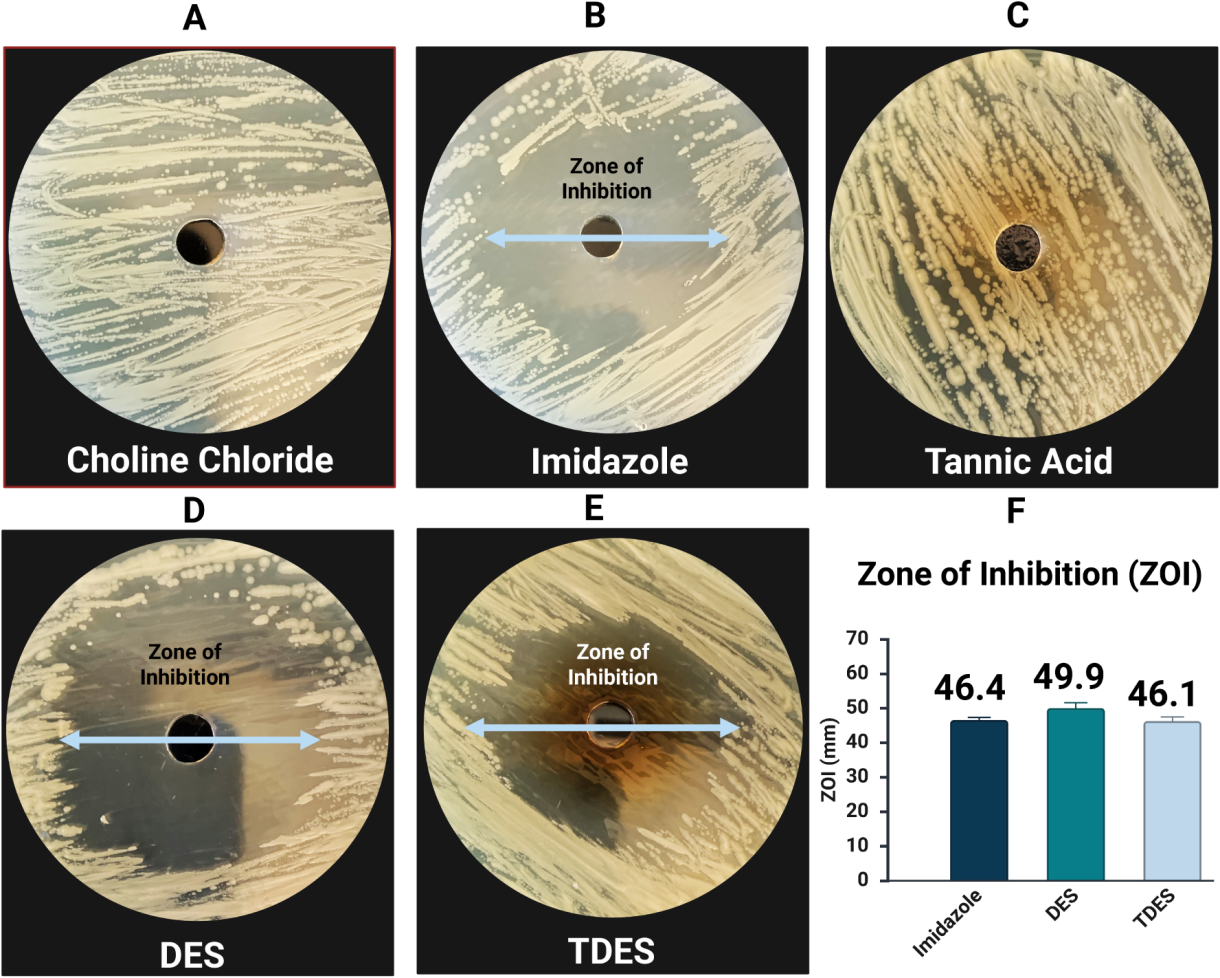
Plate–hole diffusion assay demonstrating antibacterial activity of individual components and eutectic formulations. Representative inhibition profiles of choline chloride (A), imidazole (B), and tannic acid (C), which serve as constituents of the prepared eutectic systems, are shown alongside the corresponding deep eutectic solvent (DES) (D) and ternary deep eutectic solvent (TDES) (E). Among the individual components, only imidazole produced a measurable zone of inhibition, whereas choline chloride and tannic acid showed no detectable antibacterial effect under the tested conditions. Notably, both DES and TDES exhibited clear inhibition zones, indicating eutectic formulation showing antibacterial performance beyond that of the inactive individual constituents. Quantitative comparison of zone of inhibition (ZOI) values (F) highlights the preserved or improved activity of the eutectic systems relative to imidazole alone.

The lack of antibacterial activity for choline chloride is consistent with previous studies.^18^ Its molecular structure is not membrane-active because it lacks a hydrophobic chain.^19^ Without this amphiphilic character, it cannot effectively penetrate or disrupt bacterial membranes. In addition, choline is a biologically compatible nutrient, meaning many microbes can tolerate or utilize choline-related compounds. For tannic acid, the absence of antibacterial activity at low concentrations may be explained by its limited diffusion and reduced availability in agar-based assays.^20^ Due to its relatively large molecular size and strong binding tendency,^21^ tannic acid may not spread efficiently through the agar medium. In contrast, imidazole, Figure 1B, showed clear antibacterial activity. This effect is likely related to its molecular structure, which contains nitrogen atoms within a five-membered aromatic ring. Such features can interfere with microbial cell functions and contribute to growth inhibition.^10^ Interestingly, both the deep eutectic solvent (DES) composed of choline chloride and imidazole, Figure 1D, and the ternary deep eutectic solvent (TDES) containing choline chloride, imidazole, and tannic acid, Figure 1E, exhibited antibacterial activity. This suggests that imidazole plays the dominant role, overcoming the limitations of the other components. Quantitative analysis, Figure 1F, shows that the DES system increased the ZOI by approximately 7.54%, from 46.4 mm to 49.9 mm against *E. coli*. The slight decrease observed for TDES (46.1 mm, a 0.65% reduction compared with imidazole alone) may be due to slower diffusion caused by the presence of tannic acid. Based on zone diameter interpretative standards for *E. coli*,^17^ imidazole, DES, and TDES all fall within the “susceptible” category (ZOI > 17 mm), indicating that bacterial growth was effectively inhibited. These findings support the assumption that imidazole is primarily responsible for the antibacterial behavior observed in the eutectic solvent systems.

### 3.2 Spectral Assignment of Individual Components and Eutectic Formulations

Proceeding to evaluate the molecular structure of eutectic solvent systems by using ^1^H-NMR (proton) and homonuclear correlation spectroscopy (COSY), individual components and eutectic formulations were first run. In Figure 2, peak assignments were assigned based on prior studies.^13,22–24^ We assigned the following proton chemical shifts as follows: for choline chloride: ^1^H NMR (400 MHz, D_2_O) δ 4.07 (d, *J* = 4.737 Hz, 1H), 3.53 (t, *J* = 5.173 Hz, 1H), 3.21 (s, 5H), Figure 2Ai. The COSY spectrum displays clear cross-peaks between the methylene protons, confirming the expected vicinal couplings along the −CH_2_–CH_2_– backbone of the choline cation, Figure 2Aii. For imidazole, spectral assignment is as follows: ^1^H NMR (400 MHz, D_2_O) δ 7.72 (d, *J* = 19.395 Hz, 1H), 7.08 (d, *J* = 18.835 Hz, 2H), 4.79 (s, 5H), Figure 2Bi. The COSY spectrum shows cross-peaks between the aromatic signals, confirming scalar coupling among the imidazole ring protons and supporting the expected proton connectivity within the five-membered heterocycle, Figure 2Bii. For tannic acid, spectral assignment is as follows: ^1^H NMR (400 MHz, D_2_O) δ 7.56–6.62 (m, 1H), Figure 2Ci, and a sugar proton region (3.5–5.8 ppm) consistent with a glucose core. The COSY spectrum primarily reveals correlations among glucose protons, confirming the coupled sugar spin system, while minimal aromatic cross-peaks reflect the weak coupling and substitution pattern of galloyl rings, Figure 2Cii.

**Figure 2:**
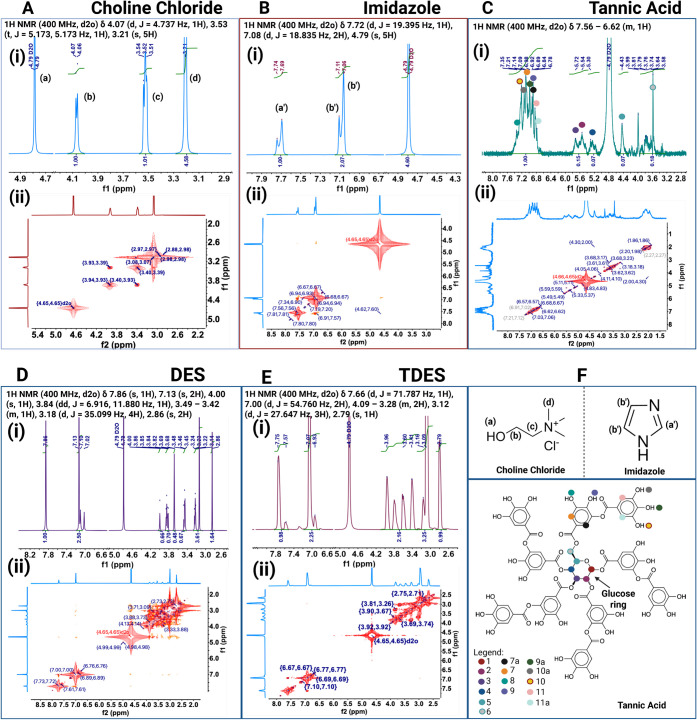
Structural verification of individual components and eutectic systems by ^1^H and COSY NMR spectroscopy. Representative ^1^H NMR spectra (i) and corresponding COSY profiles (ii) are shown for the pure components choline chloride (A), imidazole (B), and tannic acid (C), alongside the formulated deep eutectic solvent (DES) (D) and ternary deep eutectic solvent (TDES) (E). The spectra establish the chemical signatures of the individual constituents (F) and confirm their interactions within the eutectic systems through characteristic peak positions and correlation patterns. These measurements provide the essential structural baseline and compositional validation prior to biological evaluation against *E. coli* cells.

Figure 2Di spectral assignment for DES (choline chloride/imidazole) is as follows: ^1^H NMR (400 MHz, D_2_O) δ 7.86 (s, 1H), 7.13 (s, 2H), 4.00 (s, 1H), 3.84 (dd, *J* = 6.916, 11.880 Hz, 1H), 3.49–3.42 (m, 1H), 3.18 (d, *J* = 35.099 Hz, 4H), 2.86 (s, 2H). The aromatic signals near δ 7.1–7.9 ppm are from imidazole, and the aliphatic peaks between δ 2.8–4.1 ppm correspond to the choline cation. The COSY spectrum exhibits cross-peaks consistent with the expected intramolecular couplings of choline methylene groups and imidazole ring protons, confirming the coexistence of both species without evidence of new covalent bond formation, Figure 2Dii. In Figure 2Ei, spectral assignment for TDES (choline chloride/imidazole/tannic acid) is follows: ^1^H NMR (400 MHz, D_2_O) δ 7.66 (d, *J* = 71.787 Hz, 1H), 7.00 (d, *J* = 54.760 Hz, 2H), 4.09–3.28 (m, 2H), 3.12 (d, *J* = 27.647 Hz, 3H), 2.79 (s, 1H). Here, combined features of aromatic resonances (6.7–7.8 ppm) from imidazole and tannic acid together with aliphatic signals (2.7–4.1 ppm) characteristic of the choline cation were observed. The COSY spectrum shows coupling patterns consistent with the individual components, indicating preservation of their proton connectivity and suggesting that DES formation is governed by noncovalent interactions rather than new covalent bond formation, Figure 2Eii. Figure 2F summarizes the proton assignments of choline chloride, imidazole, and tannic acid, highlighting the distinct chemical environments of aliphatic (choline), heteroaromatic (imidazole), and polyphenolic/glucose (tannic acid) protons observed in the ^1^H NMR spectra.

### 3.3 Cell Collapse and Morphological Analysis

Supporting the Plate-Hole Diffusion assay results, MIC determination was tested since it is essential for identifying the lowest concentration of the eutectic solvent system that exhibits antibacterial activity. For comparative analysis, the individual components of the DES and TDES systems were also evaluated. As shown in Figure 3Ai, choline chloride (ChCl) alone slightly inhibited bacterial growth at a concentration of 22.20 mg/mL, resulting in 11.47% cell death, as presented in Figure 3Aii. In Figure 3Bi, imidazole alone exhibited minimal antibacterial activity at a concentration of 25.30 mg/mL, maintaining 93.16% cell viability, as shown in Figure 3Bii. In contrast, tannic acid alone, at a concentration of 0.051 mg/mL, induced a substantial antibacterial effect, reducing the cell viability to 16.72%, as illustrated in Figure 3Cii. The combination of choline chloride and imidazole, forming the DES system, demonstrated significantly enhanced antibacterial activity.

**Figure 3:**
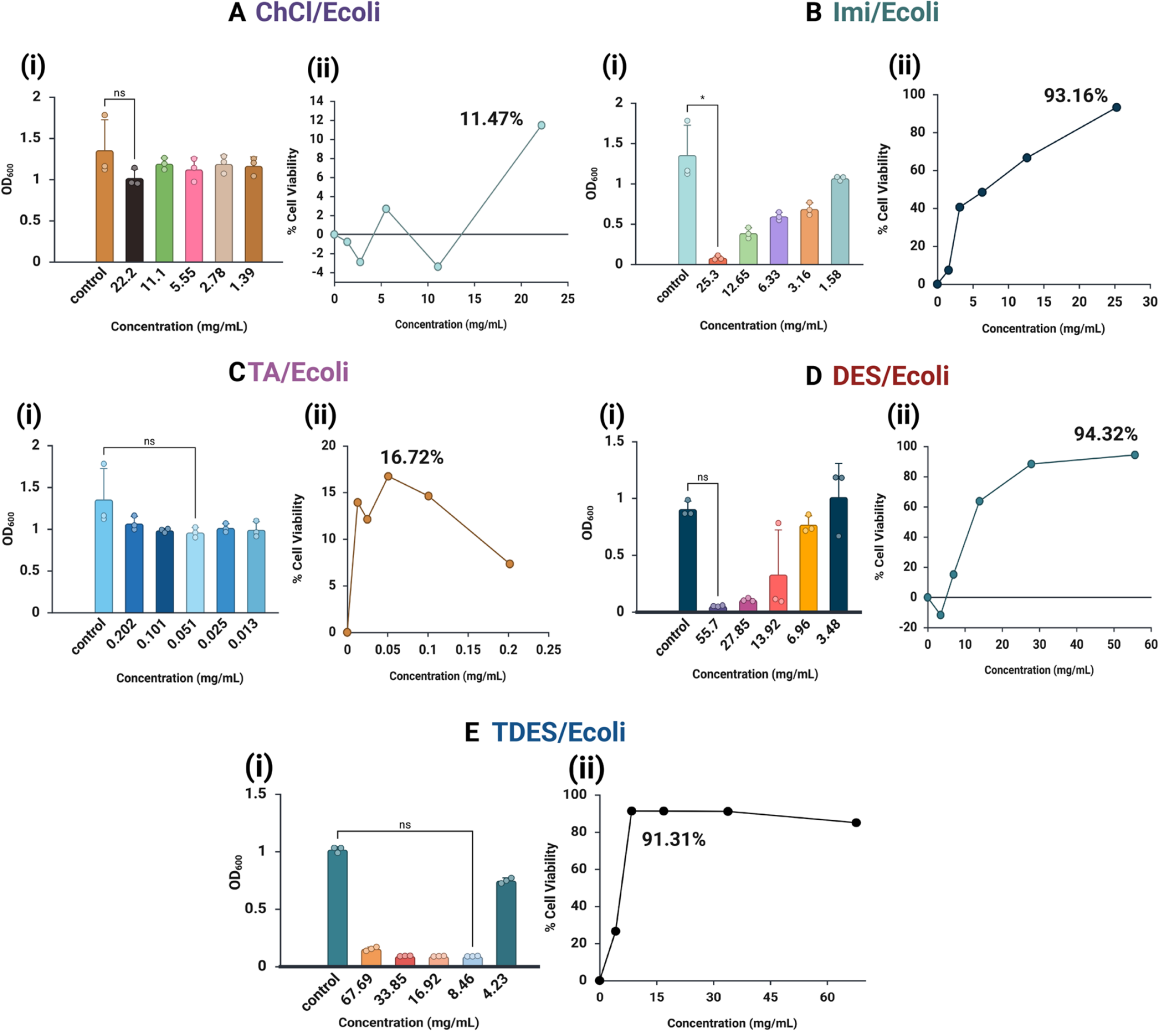
Antibacterial performance of each component: (A) Choline Chloride (ChCl). (B) Imidazole (Imi). (C) Tannic acid (TA) and its deep eutectic systems: (D) Choline Chloride/Imidazole (DES). (E) Choline Chloride/Imidazole/Tannic Acid (TDES), against *E. coli* using Minimum Inhibitory Concentration (MIC) assay and % cell viability.

As shown in Figure 3Di, the DES at a concentration of 55.70 mg/mL exhibited a clear concentration-dependent inhibitory effect against *E. coli*. Cell viability measurements further confirmed strong bactericidal activity, reaching 94.32% cell death, as presented in Figure 3Dii. The enhanced inhibition observed at higher concentrations is attributed to the increased availability of bioactive DES components. Imidazole plays a crucial role by strengthening the hydrogen-bonding network and disrupting bacterial cellular functions through its weakly basic heterocyclic structure, thereby amplifying the antibacterial performance of the eutectic system.^25^ Furthermore, TDES exhibited markedly stronger antibacterial activity than DES. As shown in Figure 3Ei, the TDES achieved significant inhibition of *E. coli* growth at a substantially lower concentration of 8.46 mg/mL, as evidenced by the pronounced reduction in the OD_600_ values. Correspondingly, cell viability analysis revealed up to 91.31% cell death, as shown in Figure 3Eii, indicating a potent antibacterial efficacy even at lower concentrations.

This enhanced efficacy compared with the DES-based system likely arises from the activation of synergistic interactions between activated tannic acid, whose polyphenolic structure can strongly interact with bacterial membranes and proteins, and the presence of imidazole, which further intensifies the effect by stabilizing the eutectic network and disrupting cellular functions, collectively leading to greater antibacterial potency at reduced concentrations. Table 2 discloses all the % cell viability measured by the concentration of each sample and its standard deviation.

SEM analysis revealed distinct morphological changes in *E. coli* cells following treatment with all samples at 1/4 × MIC concentration to emphasize the significance of the TDES results. Untreated cells, shown in Figure 4A, exhibited intact and well-defined cell walls. Cells treated with choline chloride alone, Figure 4B, and tannic acid alone, Figure 4D, primarily displayed dehydration effects associated with the fixation process, with minimal structural damage observed. In Figure 4C, cells exposed to imidazole alone showed partial cell wall collapse; however, dehydrated cells still predominated. In contrast, treatment with the DES system, Figure 4E, resulted in noticeable cell wall deformation and collapse. More severe structural disruption was observed following TDES treatment, Figure 4F, where extensive cell wall and membrane rupture were evident, confirming the enhanced antibacterial potency of the ternary eutectic system.

**Figure 4:**
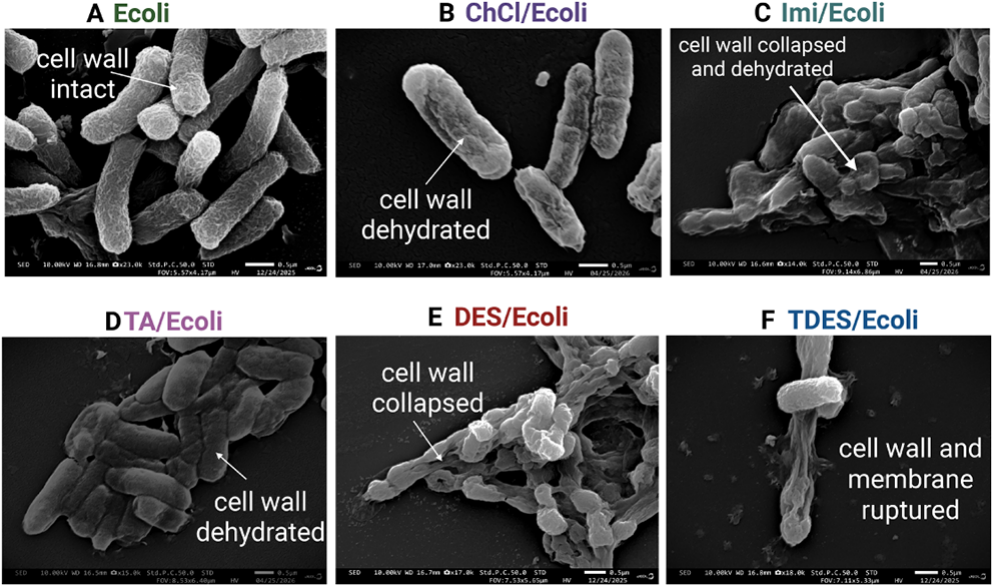
SEM micrographs, scale at 0.5 μm (A–F), corroborate MIC assay findings, revealing progressive structural damage from intact cells to cell wall collapse under DES exposure and severe cell wall and membrane rupture following TDES treatment.

Both DES and TDES demonstrated significant antibacterial activity while maintaining a strong bactericidal performance. Although imidazole alone exhibited greater antibacterial potency at a lower concentration, this difference reflects the intrinsic nature of the eutectic systems rather than a limitation of their performance. DESs and TDESs do not merely function as carriers of imidazole; instead, they operate as structured eutectic networks in which intermolecular interactions, particularly hydrogen bonding, influence the behavior and activity of the active component. The sustained antibacterial efficacy indicates that imidazole remains functionally active within the systems. Importantly, DESs and TDESs provide advantages beyond antibacterial potency alone, including tunable composition, enhanced formulation flexibility, and the potential for controlled or sustained antimicrobial activity. Therefore, despite requiring higher concentrations than pure imidazole, these eutectic systems remain highly promising multifunctional antimicrobial platforms with substantial bioactivity and physicochemical versatility that cannot be achieved by using imidazole alone.

### 3.4 ^1^H and COSY NMR Analysis of *E. coli* cells after MIC

The ^1^H NMR spectrum of the untreated *E. coli* cell extract following the MIC assay at 1/4 × MIC concentration, Figure 5A, displays characteristic metabolite resonances, indicating preservation of normal cellular biochemical composition. Prominent signals at δ 10.18 and 9.58 ppm are consistent with downfield-exchanging protons or aromatic/aldehydic-type environments,^26^ while peaks at δ 2.13, 1.91, and 0.74 ppm correspond to aliphatic metabolites,^27^ collectively reflecting intact metabolic activity and cellular integrity in the absence of DES or TDES exposure.

**Figure 5:**
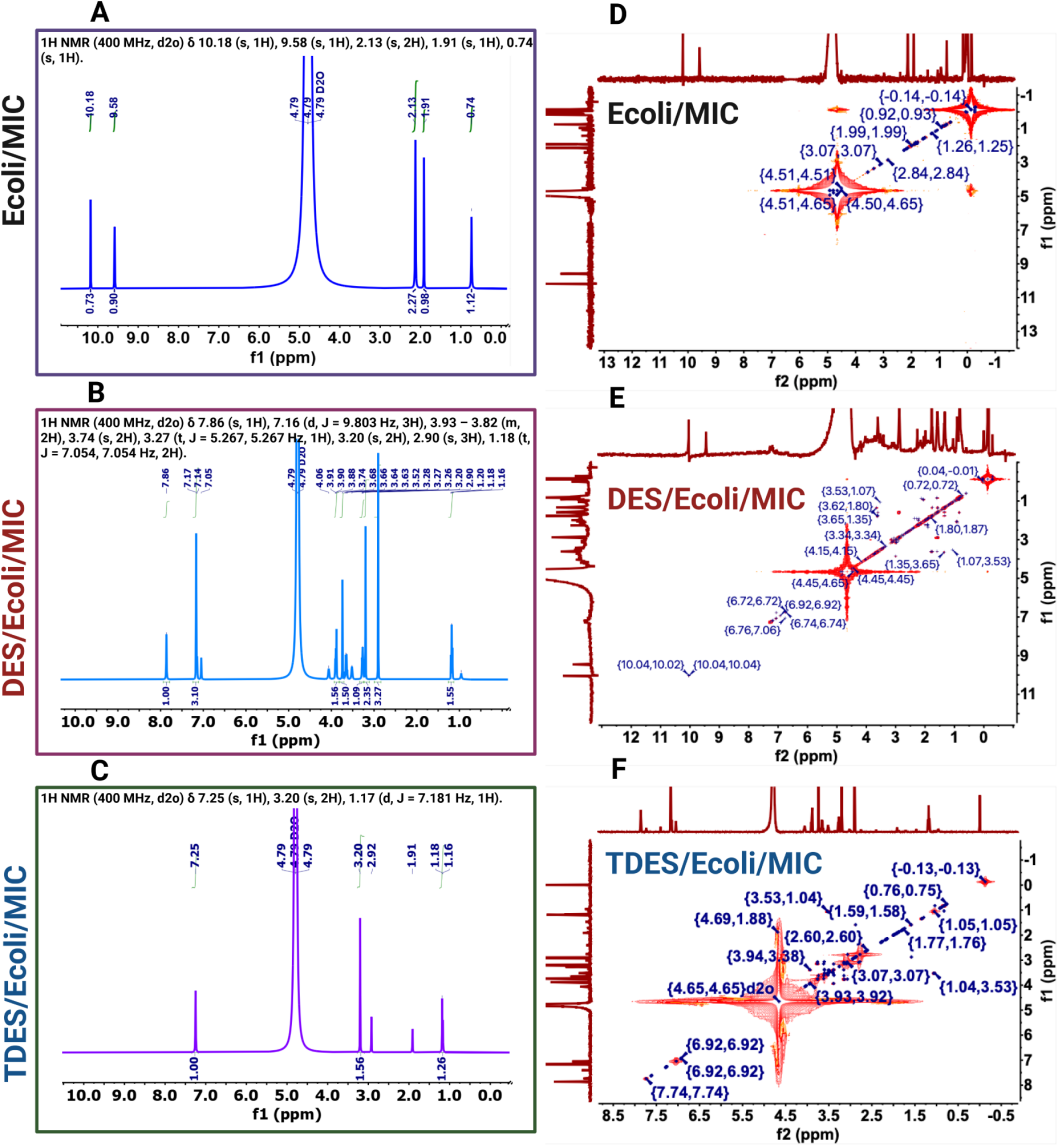
^1^H NMR spectra of *E. coli* cell extracts obtained after the MIC assay comparing untreated cells (A) with those exposed to DES (B) and TDES (C), with corresponding COSY spectra (D–F).

Compared with the untreated *E. coli* extract, the ^1^H NMR spectrum following DES exposure, Figure 5B, exhibits additional prominent signals in the aromatic region at δ 7.86 and 7.16 ppm, together with intensified aliphatic resonances between δ 3.95–3.20 ppm and δ 2.90–1.18 ppm, indicating pronounced metabolic perturbation. These spectral modifications are consistent with the contribution of imidazole in the DES, whose heterocyclic, weakly basic nature enables interactions with cellular components, disrupts proton homeostasis, and alters membrane-associated environments,^28^ thereby driving the observed biochemical and antibacterial effects. Furthermore, the TDES-exposed *E. coli* extract, Figure 5C, shows a much simpler pattern dominated by a single aromatic resonance at δ 7.27 ppm and fewer aliphatic signals at δ 3.22 ppm and δ 1.19 ppm. Compared to DES-exposed cells, peak disappearance is attributed to tannic acid altering imidazole’s behavior through strong hydrogen-bonding and π–π/ionic interactions that reduce the amount of free imidazole and broaden/shift its resonances,^29^ while the stronger antibacterial action of TDES causes more extensive metabolic depletion and/or leakage from cells, leaving fewer detectable intracellular metabolites and therefore fewer observable peaks.^30^ The COSY spectra provide mechanistic evidence that imidazole-based eutectic solvents disrupt *E. coli* metabolism primarily through membrane destabilization and intracellular biochemical perturbation.

In untreated cells, Figure 5D, correlations are dominated by well-defined aliphatic spin systems (0.9–2.0 ppm), reflecting intact pools of native metabolites, whereas DES exposure, Figure 5E, introduces prominent aromatic correlations in the 6.7–7.0 ppm region, characteristic of imidazole protons, alongside reorganized aliphatic networks, indicating metabolic stress, proton imbalance, and altered enzyme activity. These spectral changes are consistent with imidazole’s weakly basic, heteroaromatic nature, which perturbs proton homeostasis and membrane-associated processes,^31–34^ leading to the redistribution or leakage of metabolites. In the TDES system, Figure 5F, COSY patterns become markedly simplified, with fewer metabolite cross-peaks, signifying more extensive cellular damage and metabolite depletion, while retained aromatic features suggest strong participation of imidazole/tannic acid interactions likely because it enhances solvent–membrane/protein interactions and reinforces the eutectic network,^14,23^ thereby amplifying membrane disruption and accelerating metabolic collapse.

For comparative analysis, the effects of the individual components on *E. coli* cells were also investigated. As shown in Figure 6, the presence of chemical shifts at δ 10.21, Figure 6A, δ 10.18, Figure 6B, and δ 10.16, Figure 6C, corresponding to aromatic metabolites, strongly suggests why the DES and TDES systems exhibit greater antibacterial effectiveness and potential as antimicrobial agents. The detection of these aromatic metabolites indicates that a substantial number of bacterial cells remained metabolically active and may potentially develop resistance to the individual components over prolonged exposure. Furthermore, the COSY spectra shown in Figure 6D–F revealed strong proton–proton interactions within the aliphatic metabolite region (δ 1.0–4.5). These findings further support the metabolic behavior observed in the ^1^H NMR spectra of the individual components. Together, the spectral analyses suggest that while the individual components exhibit some antibacterial activity, they are less effective in disrupting bacterial metabolic processes compared to the DES and TDES systems.

**Figure 6:**
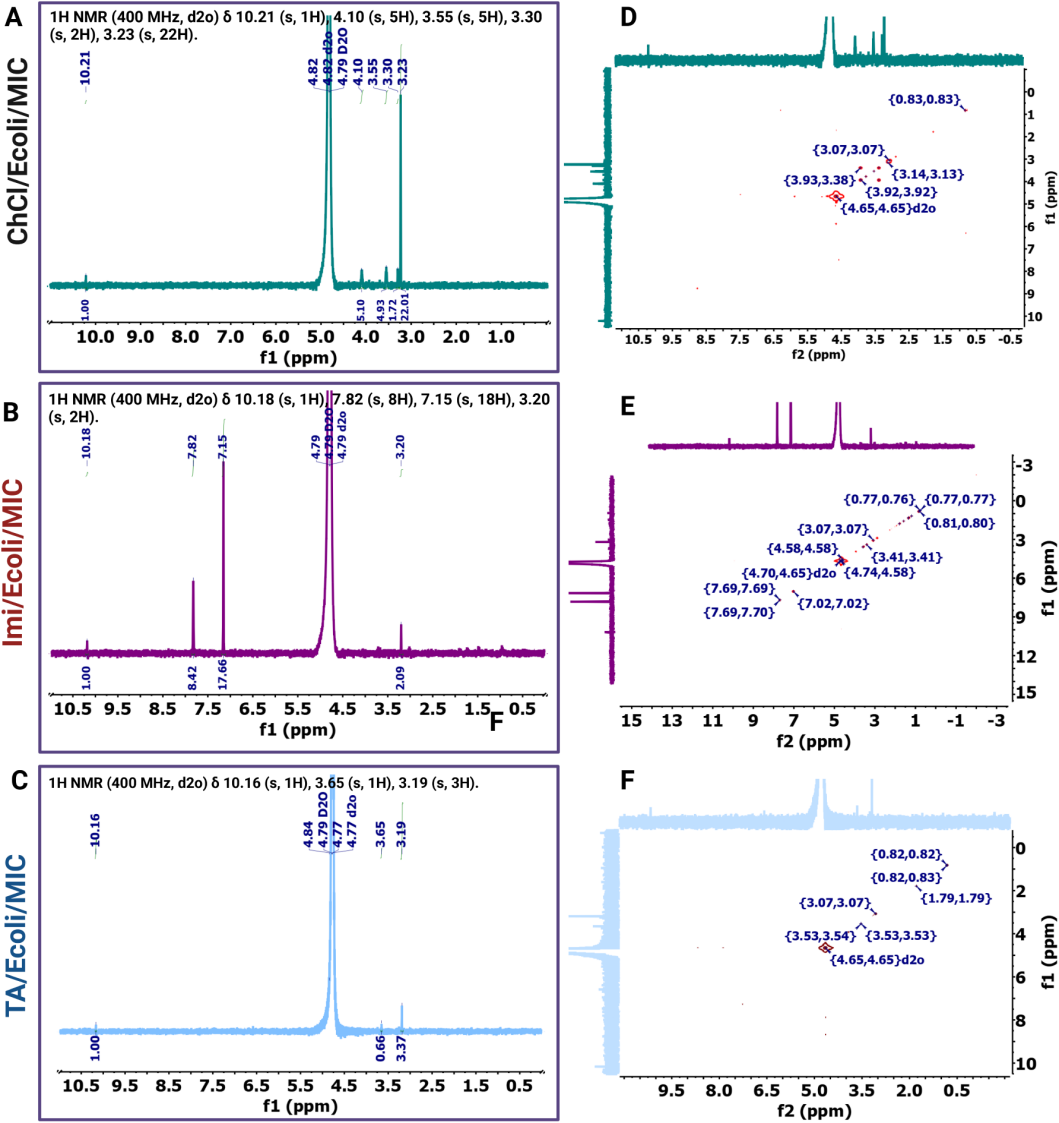
^1^H NMR with corresponding COSY NMR spectra of *E. coli* cell extracts obtained after the MIC assay exposed to individual components of the sample: Choline Chloride-ChCl (A,D); Imidazole-Imi (B,E); and Tannic Acid- TA­(C,F).

### 3.5 Oxidative Stress and Morphological Analysis

Reactive oxygen species (ROS) measurements were conducted to further elucidate the antibacterial mechanism of the eutectic systems, as oxidative stress is a well-established indicator of antimicrobial-induced cellular damage. As shown in Figure 7A, untreated *E. coli* cells exhibited low fluorescence intensity, reflecting a normal intracellular redox balance. Among the individual components, imidazole induced a pronounced increase in ROS generation, indicating a strong oxidative activity. In contrast, cells treated with the DES system exhibited a more moderate increase in fluorescence intensity, suggesting that incorporation into the eutectic network modulates the activity of the constituent components. Notably, TDES-treated cells displayed the highest fluorescence intensity among all the groups, indicating severe oxidative stress and enhanced antibacterial activity.

**Figure 7:**
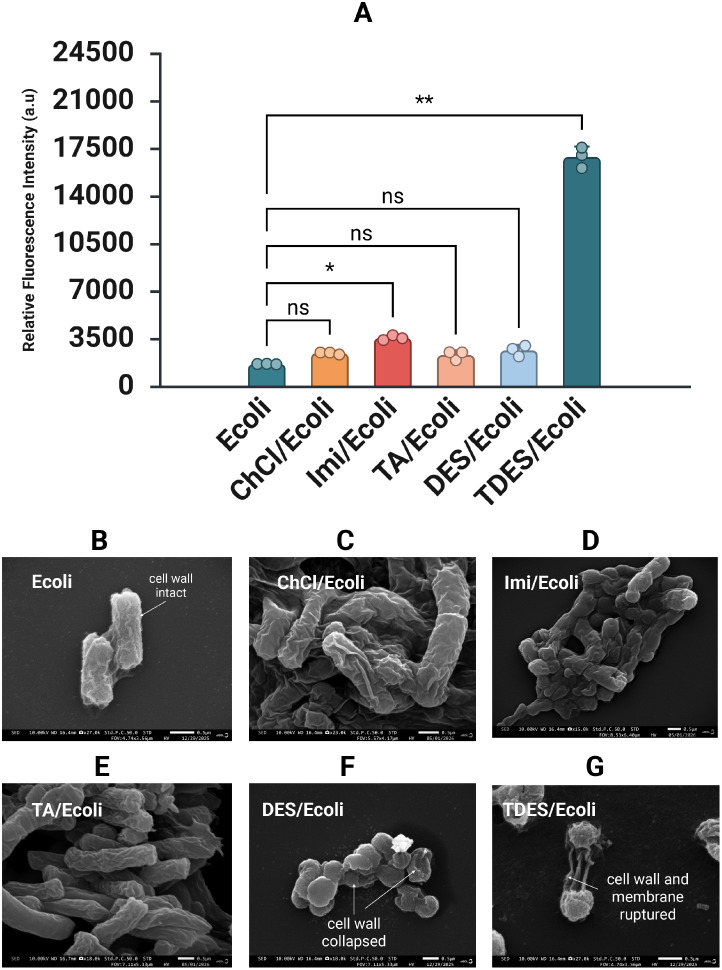
Performance of the eutectic systems against *E. coli* from an ROS perspective. ROS analysis (A) reveals treatment-dependent oxidative stress, where DES induces a moderate increase in fluorescence intensity and TDES produces a markedly amplified ROS response, indicating severe oxidative damage. SEM micrographs, scale at 0.5 μm (B–G), corroborate these observations.

The SEM images further supported these observations by revealing morphological changes that were correlated with the measured ROS levels. Untreated cells, Figure 7B, retained intact morphology with smooth and well-defined cell walls. Cells treated with choline chloride, Figure 7C, and imidazole, Figure 7D, exhibited surface irregularities primarily associated with dehydration during the fixation process, although some imidazole-treated cells showed visible structural damage. In comparison, tannic acid-treated cells, as shown in Figure 7E, demonstrated relatively milder morphological alterations.

More pronounced structural damage was observed in cells treated with the DES system (Figure 7F), which exhibited noticeable cell wall collapse, indicating a compromised membrane integrity. In contrast, TDES-treated cells, Figure 7G, displayed extensive structural disruption, including rupture of both the cell wall and cellular membrane. These findings collectively indicate that oxidative stress plays a major role in bacterial cell damage. Although imidazole alone acts as a strong inducer of ROS, its incorporation into eutectic systems alters its oxidative behavior, resulting in a moderated response in DESs and a substantially amplified effect in TDESs, likely due to synergistic intermolecular interactions within the ternary eutectic system.

### 3.6 ^1^H and COSY NMR Analysis of *E. coli* cells after ROS

The ^1^H NMR spectra of *E. coli* cell extracts following the ROS assay provide molecular-level insight into how imidazole-based DES exposure alters cellular metabolism under oxidative stress conditions. In the untreated, Figure 8A, the spectrum shows a balanced distribution of aliphatic resonances typical of native intracellular metabolites, reflecting preserved metabolic activity and redox homeostasis.

**Figure 8:**
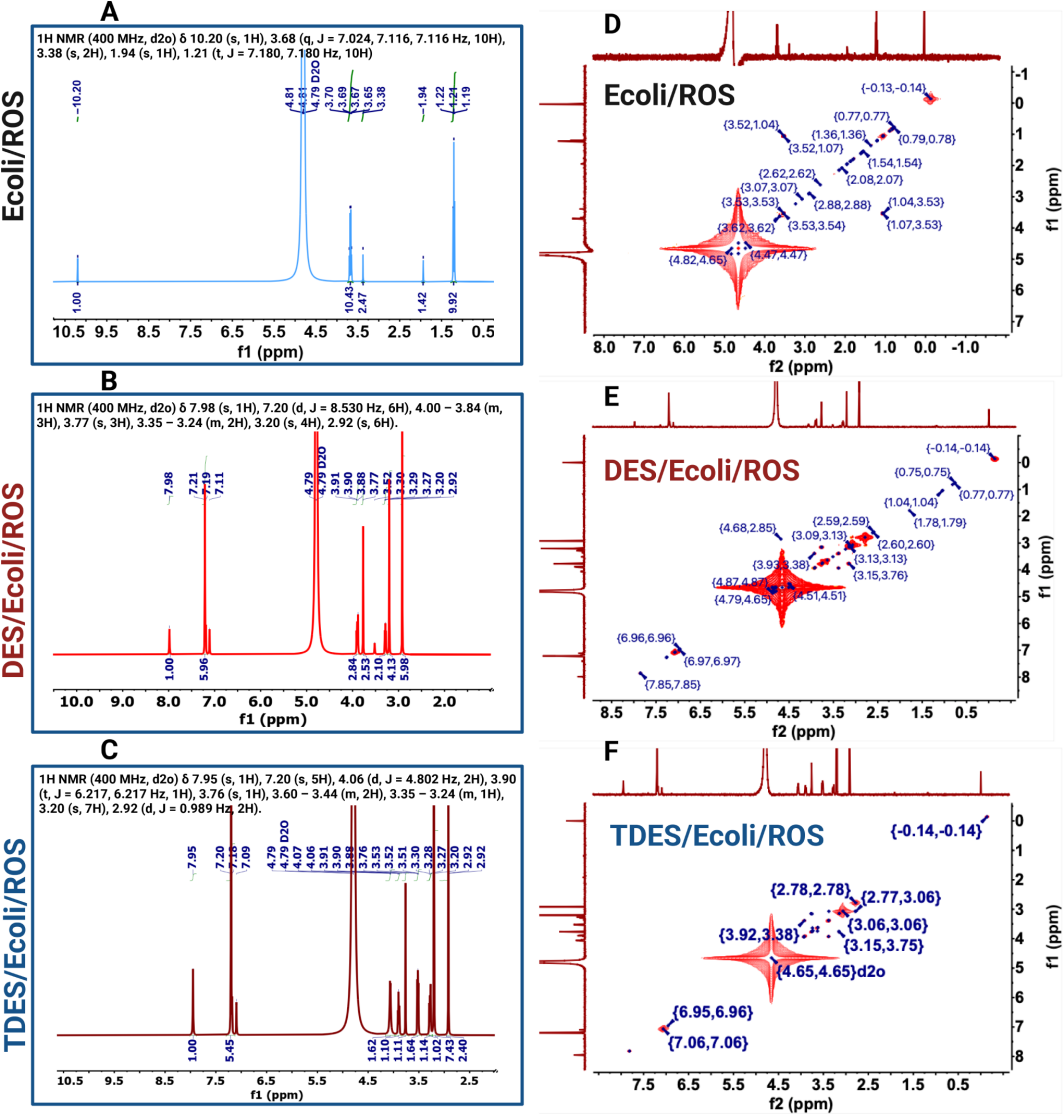
^1^H NMR spectra of *E. coli* cell extracts obtained after the ROS assay comparing untreated cells (A) with those exposed to DES (B) and TDES (C), with corresponding COSY spectra (D–F).

Upon DES treatment, Figure 8B, new and intensified aromatic resonances appear in the δ 7.18–7.96 ppm region, consistent with imidazole-associated protons, accompanied by pronounced modifications in the δ 3.92–3.18 ppm range, corresponding to methylene-rich metabolites and small polar molecules. These changes indicate that DES exposure perturbs the intracellular chemical environment, likely through imidazole-driven interactions that disrupt proton balance and enzyme-mediated pathways.^35^ Imidazole’s heterocyclic, weakly basic character is particularly significant, as it can interfere with membrane-associated processes and intracellular pH regulation, thereby promoting ROS formation. Elevated ROS levels, in the TDES-exposed sample, induce oxidative damage to lipids, proteins, and metabolic intermediates, leading to metabolite depletion, redistribution,^36,37^ or altered mobility, which manifest in the NMR spectra as peak broadening and intensity variation, as observed in Figure 8C.

The COSY spectra of *E. coli* cell extracts following the ROS assay reveal pronounced treatment-dependent changes in proton coupling networks, providing insight into metabolic disruption^38,39^ induced by the imidazole-based eutectic systems. In the untreated sample, Figure 8D, the COSY map displays well-defined cross-peaks predominantly within the aliphatic region (1.0–4.0 ppm), consistent with intact coupling patterns of native intracellular metabolites and normal metabolic connectivity. After DES exposure, Figure 8E, additional correlations emerge alongside altered cross-peak intensities, particularly involving resonances near δ 3.1–3.9 ppm and aromatic signals around δ 7.8–7.9 ppm, indicating perturbation of small polar metabolites and the presence of imidazole-associated spin systems. Elevated ROS levels can damage membrane structures and enzyme function, leading to metabolite leakage or altered intracellular dynamics, which manifest as modified or weakened COSY correlations. In contrast, the TDES-treated extract, Figure 8F, shows a markedly simplified COSY pattern with fewer metabolite cross-peaks, highlighting the amplified stress effects arising from synergistic interactions within the ternary eutectic system. The spectral differences demonstrate that oxidative stress is not merely a secondary effect but a central mechanism of DES-induced antibacterial activity, with imidazole acting as a key mediator linking solvent structure to metabolic and redox disruption.

For comparative analysis, the effects of the individual components on *E. coli* cells were also evaluated using ROS assays. Cells treated with choline chloride, Figure 9A, exhibited relatively simple spectral features, indicating limited metabolic disruption. In contrast, imidazole-treated cells, Figure 9B, displayed more complex and intense spectral signals, suggesting significant alterations in intracellular metabolites consistent with elevated oxidative stress. Tannic acid-treated cells, Figure 9C, showed a more defined yet less complex spectral profile, indicating a moderate metabolic response. The corresponding COSY spectra, Figure 9D–F, further supported these observations by revealing distinct differences in proton–proton coupling patterns among the treatments. Imidazole-treated samples, Figure 9E, exhibited a higher density of cross-peaks, reflecting greater molecular diversity and more pronounced metabolic perturbation. In comparison, choline chloride-treated, Figure 9D, and tannic acid-treated samples, Figure 9F, displayed relatively simpler interaction patterns, suggesting less extensive intracellular disruption. These findings indicate that imidazole induces more substantial intracellular metabolic changes, consistent with its stronger involvement in ROS-mediated oxidative stress, whereas choline chloride and tannic acid produce comparatively moderate effects on bacterial metabolism.

**Figure 9:**
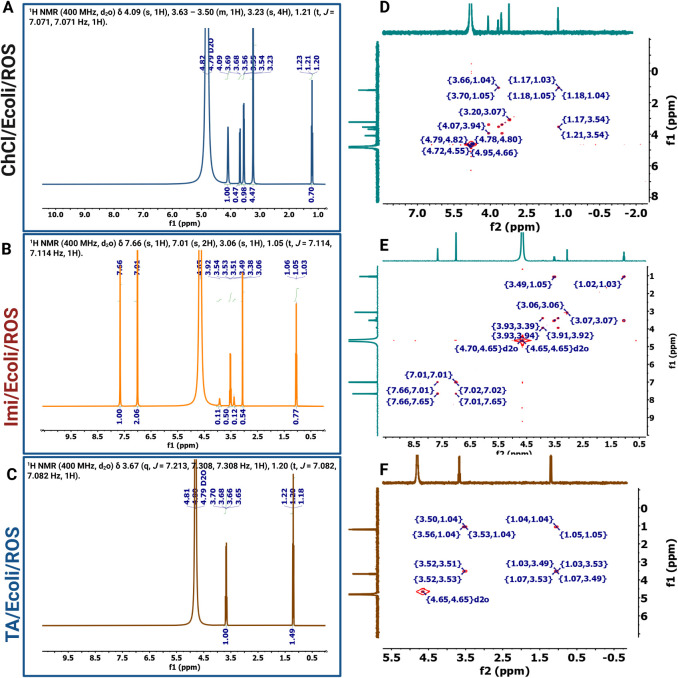
^1^H NMR spectra of *E. coli* cell extracts obtained after the ROS assay comparing treated cells (A) with those exposed to individual components of DES and TDES: Choline Chloride (ChCl) (A). Imidazole (Imi) (B). Tannic Acid (TA) (C), with corresponding COSY spectra (D–F).

## 4 Conclusion

This study establishes that imidazole acts as a molecular activity switch capable of converting eutectic solvents into highly effective antibacterial systems against *Escherichia coli* where the combined MIC, ROS, and SEM results demonstrate that imidazole-enabled DES and TDES induce distinct but related killing phenotypes, ranging from cell wall collapse to catastrophic wall–membrane rupture. Complementarily, NMR spectroscopy provides the critical molecular-level interpretation linking these biological outcomes to changes in chemical environments and metabolite networks. Herein, highlighting the ^1^H and COSY NMR data reveals that antibacterial activity arises from the direct observation of proton connectivity, metabolite redistribution, and solvent–cell interactions that are otherwise inaccessible through conventional assays alone. Thus, NMR suggests as an emerging analytical technique for biological studies by detecting subtle metabolic perturbations and differentiating between membrane damage, oxidative stress effects, and component-specific interactions. These findings underscore the value of NMR spectroscopy as a predictive and explanatory platform for antimicrobial research, offering a robust analytical bridge between physicochemical solvent behavior and biological function and supporting the rational design of next-generation imidazole-activated eutectic antimicrobial systems.

## References

[1] Kavanagh K. (2025). The rise of
‘nightmare bacteria’: antimicrobial resistance in five
charts. Nature.

[2] Karim M., KianvashRad N., Cabo M., Adegoke S. C., Tuffour K., Duah R., Yawlui I. S. Y., Lajeunesse D. (2026). Cell Adhesion
and Biofilm Development via Force-Sensitive Mechanisms: A Perspective. ACS Biomater. Sci. Eng..

[3] Swebocki T., Kocot A. M., Cieminska K., Bortolus C., Muchembled J., Mechouche M. S., Jacquin J., Haddadi K., Siah A., Sendid B., Boukherroub R., Plotka M. (2025). Breaking through Microbial
Defenses—Organic Acid-Based Deep Eutectic Solvents as a Neoteric
Strategy in Bacterial Biofilms, Persister, and Fungal Control. ACS Appl. Bio Mater..

[4] Subramani R. M., Shamprasad B. R., Viswanathan N. S. S. (2024). Therapeutic deep eutectic
solvent with saponin, optimized through response surface methodology,
exert potent in vivo antimicrobial effects against *Pseudomonas
aeruginosa*. Sci. Rep..

[5] Tjalsma T. G., Didion Y. P., Su Z., Malankowska M., Torres-Montero P., Martínez J. L., Pinelo M. (2025). Hydrophobic deep eutectic
solvents as novel, sustainable aids for intracellular protein release
from Saccharomyces cerevisiae. Results Eng..

[6] Popović B. M., Gligorijević N., Aranđelović S., Macedo A. C., Jurić T., Uka D., Mocko-Blažek K., Serra A. T. (2023). Cytotoxicity profiling
of choline chloride-based natural
deep eutectic solvents. RSC Adv..

[7] Cajnko M. M., Vicente F. A., Novak U., Likozar B. (2023). Natural deep eutectic
solvents (NaDES): translating cell biology to processing. Green Chem..

[8] Mohammad F., Azizi N., Mirjafari Z., Mokhtari J. (2025). Green synthesis of
imidazole derivatives in a ternary deep eutectic solvent system. Sci. Rep..

[9] Duan Y. T., Wang Z. C., Sang Y. L., Tao X. X., Zhu H. L. (2013). Exploration
of Structure-Based on Imidazole Core as Antibacterial Agents. Curr. Top. Med. Chem..

[10] Hu Y., Xing Y., Ye P., Yu H., Meng X., Song Y., Wang G., Diao Y. (2023). The antibacterial activity
and mechanism of imidazole chloride ionic liquids on Staphylococcus
aureus. Front. Microbiol..

[11] Moghadam F. A., Dabirian S., Tavani A. E., Alipour P., Mojabi M., Evazalipour M., Yousefbeyk F., Ghasemi S. (2024). Application of a Deep
Eutectic Solvent for the Synthesis of Novel Imidazole-Containing Quinazoline
Derivatives as Potent Cytotoxic Agents. Pharm
Sci..

[12] Mjalli F. S., Shakourian-Fard M., Kamath G., Murshid G., Naser J., Al Ma’awali S. (2023). Experimental and theoretical study of the physicochemical
properties of the novel imidazole-based eutectic solvent. J. Mol. Graphics Modell..

[13] Muzio S. D., Russina O., Mastrippolito D., Benassi P., Rossi L., Paolone A., Ramondo F. (2022). Mixtures of
choline chloride and
tetrabutylammonium bromide with imidazole as examples of deep eutectic
solvents: their structure by theoretical and experimental investigation. J. Mol. Liq..

[14] Cabo M., Kattel S., LaJeunesse D. (2025). Tuning Ternary Deep Eutectic Solvent
Semiconductivity and Specific Capacitance Properties via Solubilizing
Bacterial Nanocellulose for Flexible Soft Material. ACS Mater. Au.

[15] MacWilliams, M. P. ; Liao, M. K. Luria Broth (LB) and Luria Agar (LA) Media and Their Uses Protocol; American Society for Microbiology, 2006. https://asm.org/getattachment/5d82aa34-b514-4d85-8af3-aeabe6402874/lb-luria-agar-protocol-3031.pdf. Accessed: Feb. 12, 2026.

[16] Hayhoe, E. J. ; Palombo, E. A. Screening for Antibacterial, Antifungal, and Anti quorum Sensing Activity. In Metabolomics Tools for Natural Product Discovery, Roessner, U. ; Dias, D. , Eds., Methods in Molecular Biology; Humana Press: Totowa, NJ, 2013, Vol. 1055; pp. 219–225. DOI: 10.1007/978-1-62703-577-4_16. 23963914

[17] Hudzicki, J. Kirby-Bauer Disk Diffusion Susceptibility Test Protocol; American Society for Microbiology, 2009. https://asm.org/getattachment/2594ce26-bd44-47f6-8287-0657aa9185ad/kirby-bauer-disk-diffusion-susceptibility-test-protocol-pdf.pdf. Accessed. Feb. 12, 2026.

[18] Radošević K., Bubalo M. C., Srček V. G., Grgas D., Dragičević T. L., Redovniković I. R. (2015). Evaluation of toxicity and biodegradability
of choline chloride based deep eutectic solvents. Ecotoxicol. Environ. Saf..

[19] Choline Chloride; PubChem, https://pubchem.ncbi.nlm.nih.gov/compound/Choline-Chloride. Accessed: Feb. 12, 2026.

[20] Hossain T.
J. (2024). Methods
for screening and evaluation of antimicrobial activity: A review of
protocols, advantages, and limitations. Eur.
J. Microbiol. Immunol..

[21] Chen C., Yang H., Yang X., Ma Q. (2022). Tannic acid: a crosslinker
leading to versatile functional polymeric networks: a review. RSC Adv..

[22] Delso I., Lafuente C., Muñoz-Embid J., Artal M. (2019). NMR study of choline
chloride-based deep eutectic solvents. J. Mol.
Liq..

[23] Cabo M., Ibrahim A. A., Pathiraja G., Ebrahimi F., Mantripragada S., Ayoub O., Khader B., Dellinger K., Alston J. R., Obare S. O. (2026). Transmission
Electron Microscopy for Structural Insights into Bacterial Cellulose
Nanowhiskers in Ternary Deep Eutectic Solvent. ACS Meas. Sci. Au.

[24] Sen K., Sharma P., Chauhan K. (2020). Chloroacetyl-Mediated
Modification
of Chitosan by Tannic Acid to Synthesize Economical Tanninate-Chitosan
and Its Use in Fluoride Ions Adsorption from Aqueous Solution. ChemistrySelect.

[25] Avirdi E., Paumo H. K., Kamdem B. P., Singh M. B., Kumari K., Katata-Seru L., Bahadur I. (2023). Imidazolium-Based Ionic Liquid-Assisted
Silver Nanoparticles and Their Antibacterial Activity: Experimental
and Density Functional Theory Studies. ACS Omega.

[26] Falzone C. J., Benkovic S. J., Wright P. E. (1990). Partial
proton NMR assignments of
the *Escherichia coli* dihydrofolate reductase complex
with folate: evidence for a unique conformation of bound folate. Biochemistry.

[27] Ikeya T., Hanashima T., Hosoya S. (2016). Improved in-cell structure
determination of proteins at near-physiological concentration. Sci. Rep..

[28] Hu F., Zhang L., Nandakumar K. S., Cheng K. (2021). Imidazole Scaffold
Based Compounds in the Development of Therapeutic Drugs. Curr. Top. Med. Chem..

[29] Matthews R. P., Welton T., Hunt P. A. (2015). Hydrogen
bonding and π–π
interactions in imidazolium-chloride ionic liquid clusters. Phys. Chem. Chem. Phys..

[30] Jung D., Jung J. B., Kang S., Li K., Hwang I., Jeong J. H., Kim H. S., Lee J. (2021). Toxico-metabolomics
study of a deep eutectic solvent comprising choline chloride and urea
suggests in vivo toxicity involving oxidative stress and ammonia stress. Green Chem..

[31] Li S. R., Tan Y. M., Zhang L., Zhou C. H. (2023). Comprehensive Insights
into Medicinal Research on Imidazole-Based Supramolecular Complexes. Pharmaceutics.

[32] Lam P.-L., Wong R. S.-M., Lam K.-H., Hung L.-K., Wong M.-M., Yung L.-H., Ho Y.-W., Wong W.-Y., Hau D. K.-P., Gambari R. (2020). The role of reactive oxygen species in
the biological activity of antimicrobial agents: An updated mini review. Chem.-Biol. Interact..

[33] Chen Y.-S., Tian H.-X., Rong D.-C., Wang L., Chen S., Zeng J., Xu H., Mei J., Wang L.-Y., Liou Y.-L. (2025). ROS homeostasis in cell
fate, pathophysiology, and
therapeutic interventions. Mol. Biomed..

[34] Liu Y., Wang D., Lai Y., Zou J., Yang P., Wu Z., He W. (2024). Deep Eutectic Solvents
for Essential-Oil Delivery and
Bacterial-Infected Wound Healing. Langmuir.

[35] Shitov D. A., Krutin D. V., Tupikina E. Y. (2024). Mutual
influence of non-covalent
interactions formed by imidazole: A systematic quantum-chemical study. J. Comput. Chem..

[36] Dubinenko G., Senkina E., Golovina K., Myshova A., Igumnova O., Plotnikov E., Badaraev A., Rutkowski S., Filimonov V., Tverdokhlebov S. (2026). Tailorable Antibacterial Activity
and Biofilm Eradication Properties of Biocompatible α-Hydroxy
Acid-Based Deep Eutectic Solvents. Pharmaceutics.

[37] Ibrahim A., Tshibangu M. M., Coquelet C., Espitalier F. (2025). Ternary Choline
Chloride-Based Deep Eutectic Solvents: A Review. ChemEngineering.

[38] Martineau E., Dumez J.-N., Giraudeau P. (2020). Fast quantitative
2D NMR for metabolomics
and lipidomics: A tutorial. Magnetic Reson in Chemistry. Magn. Reson. Chem..

[39] LibreTexts Chemistry 5.1: COSY Spectra; https://chem.libretexts.org/Bookshelves/General_Chemistry/Book%3A_Structure_and_Reactivity_in_Organic_Biological_and_Inorganic_Chemistry_(Schaller)/Structure_and_Reactivity_in_Organic_Biological_and_Inorganic_Chemistry_II%3A_Practical_Aspects_of_Structure_-_Purification_and_Spectroscopy/05%3A_2D_NMR/5.01%3A_COSY_Spectra. Accessed: Feb. 15, 2026.

